# Supplementary material for: Defining spatial nonuniformities of all ipRGC types using an improved Opn4cre recombinase mouse line
Source: Cell Rep Methods. 2024 Aug 9;4(8):100837. doi: 10.1016/j.crmeth.2024.100837 (PMC11384080; doi:10.1016/j.crmeth.2024.100837)
Supplement: Document S1. Figures S1–S7 [file mmc1.pdf]

Cell Reports Methods, Volume *4*

Supplemental information

Defining spatial nonuniformities of all ipRGC types using an improved *Opn4^{cre}* recombinase mouse line

Brannen Dyer, Sue O. Yu, R. Lane Brown, Richard A. Lang, and Shane P. D'Souza

SUPPLEMENTAL FIGURES & LEGENDS

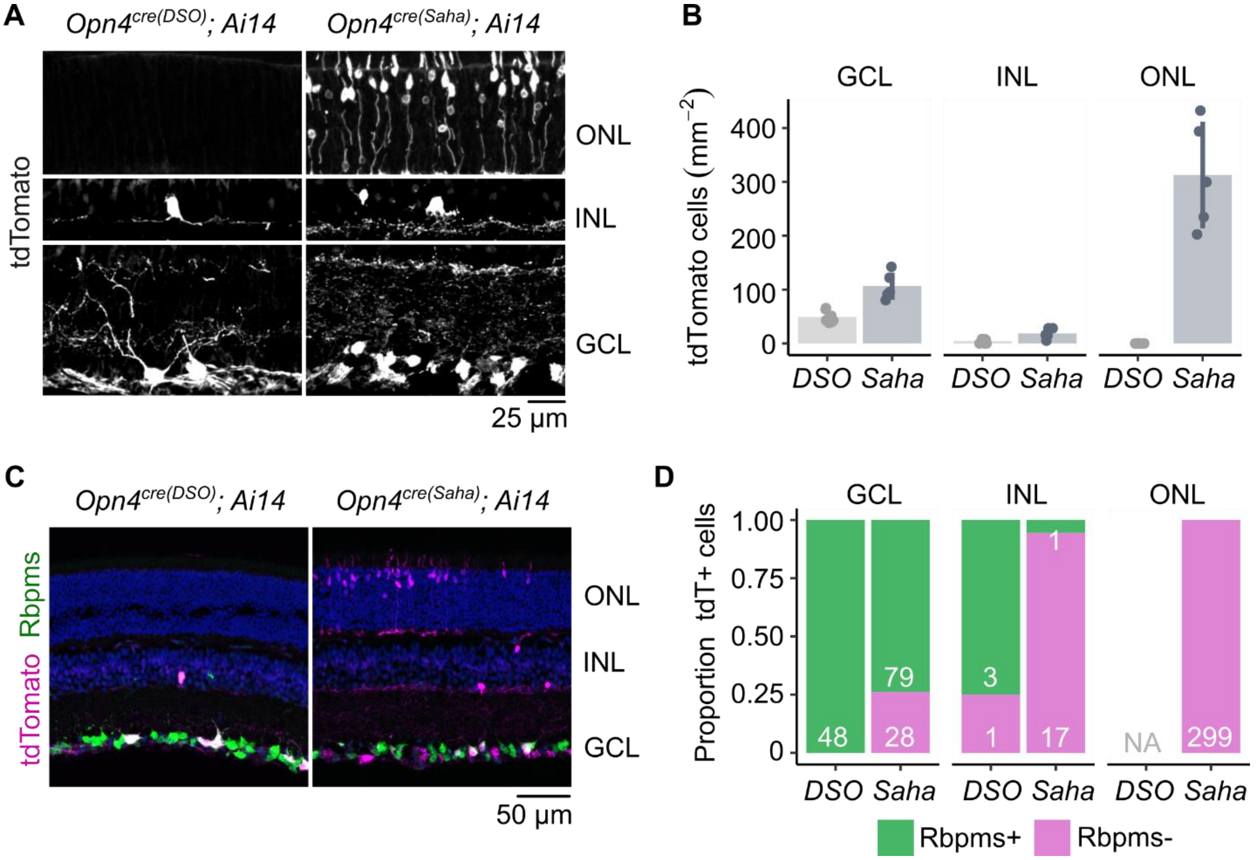

Supplemental Figure 1 | Labeled cell densities and retinal ganglion cell specificities across retinal nuclear layers, related to Figure 2.

(A) Representative confocal images highlighting tdTomato+ cells within the GCL, INL, and ONL between two *Cre* lines. **(B)** Quantification of densities (tdTomato+ cells / area) of images like in **(A)**. **(C)** Representative confocal images highlighting tdTomato+ and Rbpms+ cells within the GCL, INL, and ONL between two *Cre* lines. **(D)** Stacked bar graphs representing percentage of tdTomato cells that are Rbpms+ (green) and Rbpms- (magenta), numbers in each bar indicate number of cells surveyed. Bar height = means, error bars = standard deviations. GCL = ganglion cell layer, INL = inner nuclear layer, ONL = outer nuclear layer. Animal age = Postnatal day 60 (P60).

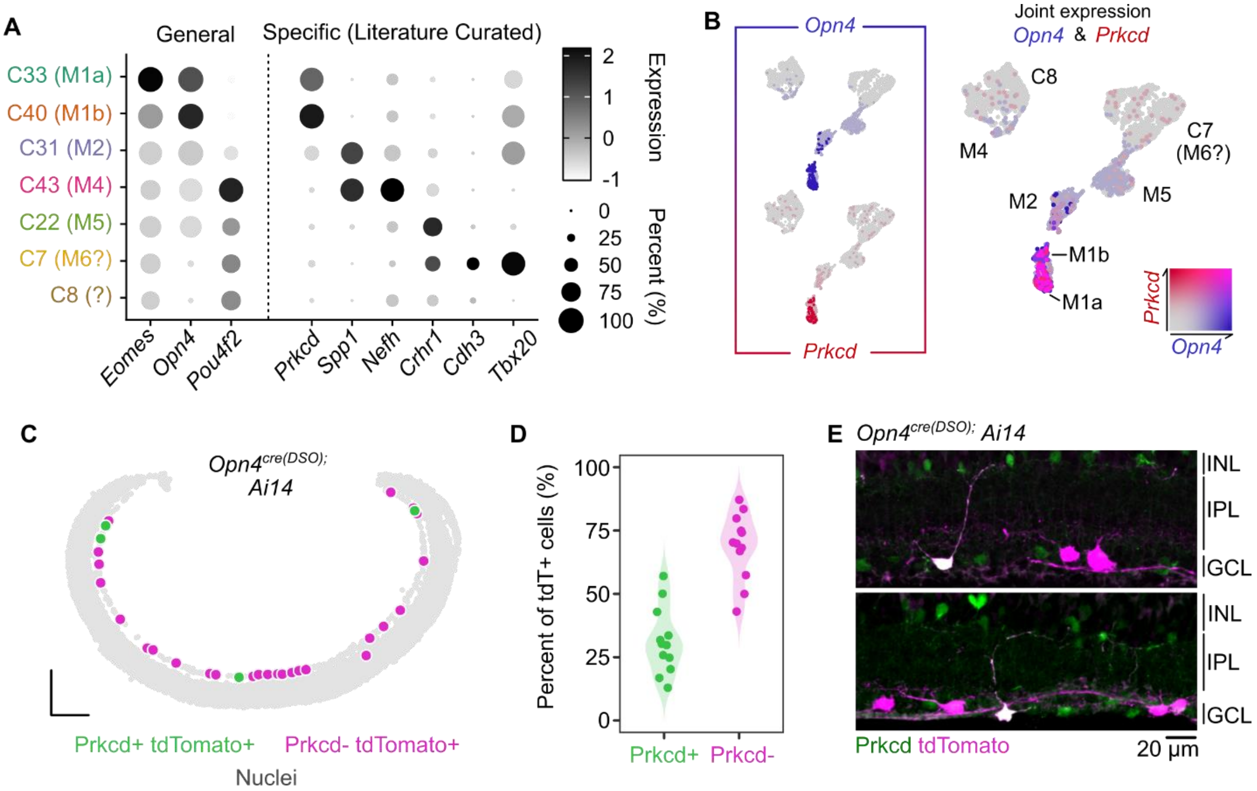

Supplemental Figure 2 | Assessment of ipRGC and ipRGC-proximal cluster markers from Tran et al., 2019, related to Figure 3.

(A) Dot-plot of general ipRGC genes (*Eomes, Opn4*, *Pou4f2*) and literature curated ipRGC type enriched genes: *Prkcd*, *Spp1*, *Nefh, Crhr1, Cdh3, Tbx20*. **(B)** UMAP embedding of *Opn4* and *Prkcd* expression across ipRGCs from Tran et al., 2019. **(C)** Graphical section representation of single- and double-labeled cells from *Opn4cre(DSO); Ai14* retinae stained with Prkcd antibodies. **(D)** Percent of tdTomato+ cells that label with Prkcd (*n* = 12 fields from 3 animals). **(E)** Representative images of S1-laminating ipRGCs (M1) strongly expressing Prkcd. Animal age = Postnatal day 60 (P60).

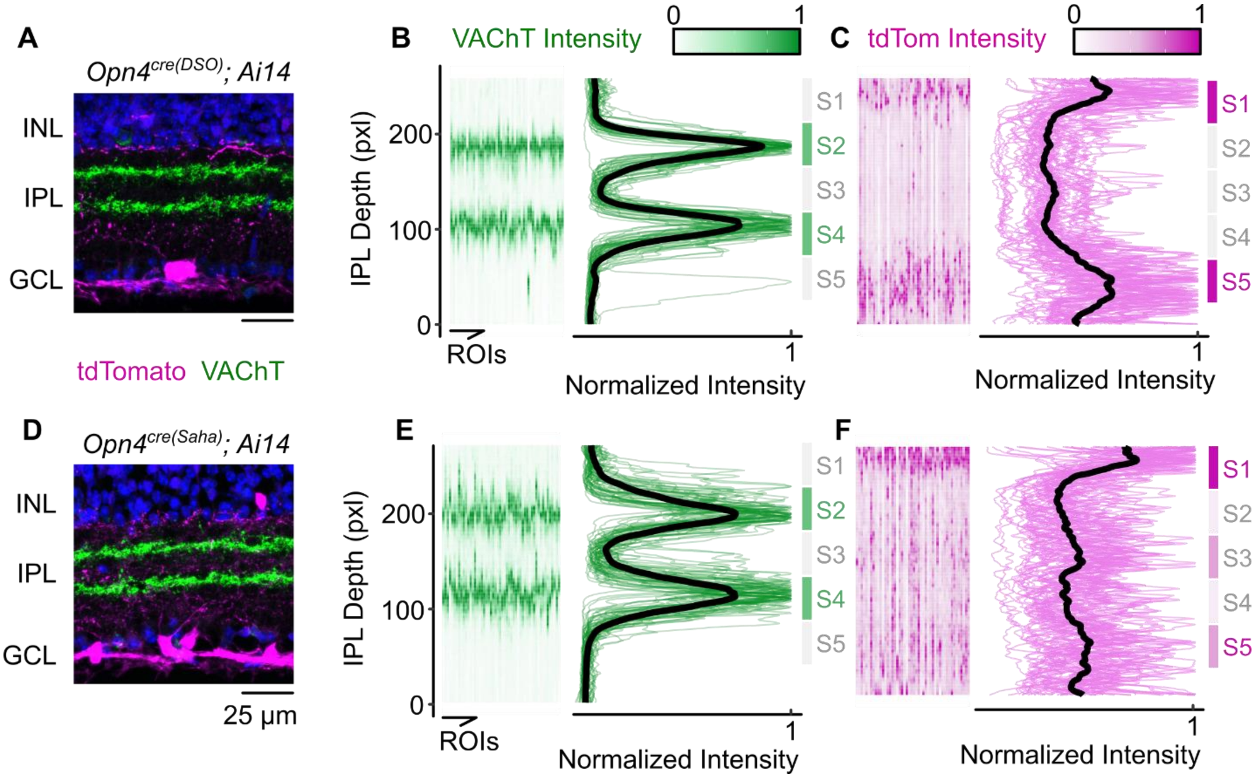

Supplemental Figure 3 | Dendritic lamination within the plexiform layer in both *Cre* lines, related to Figure 3.

(A-C) Analysis of VAChT and tdTomato signal intensity across IPL depth in the *Opn4*$^{cre(DSO)}$ line crossed to *Ai14*. VAChT marks starburst amacrine cells (SACs) that laminate in sublamina 2 and 4 (S2 & S4), and is used as an internal control for analysis. **(B)** Individual 35 x 55 µm ROIs plotted as an intensity heatmap normalized to the maximum intensity value (left) and corresponding average pixel intensity line graph depicting lamination of individual cell types. **(D-F)** Same as **(A-C)** but in the *Opn4*$^{cre(Saha)}$ line crossed to *Ai14*. Animal age = Postnatal day 60 (P60).

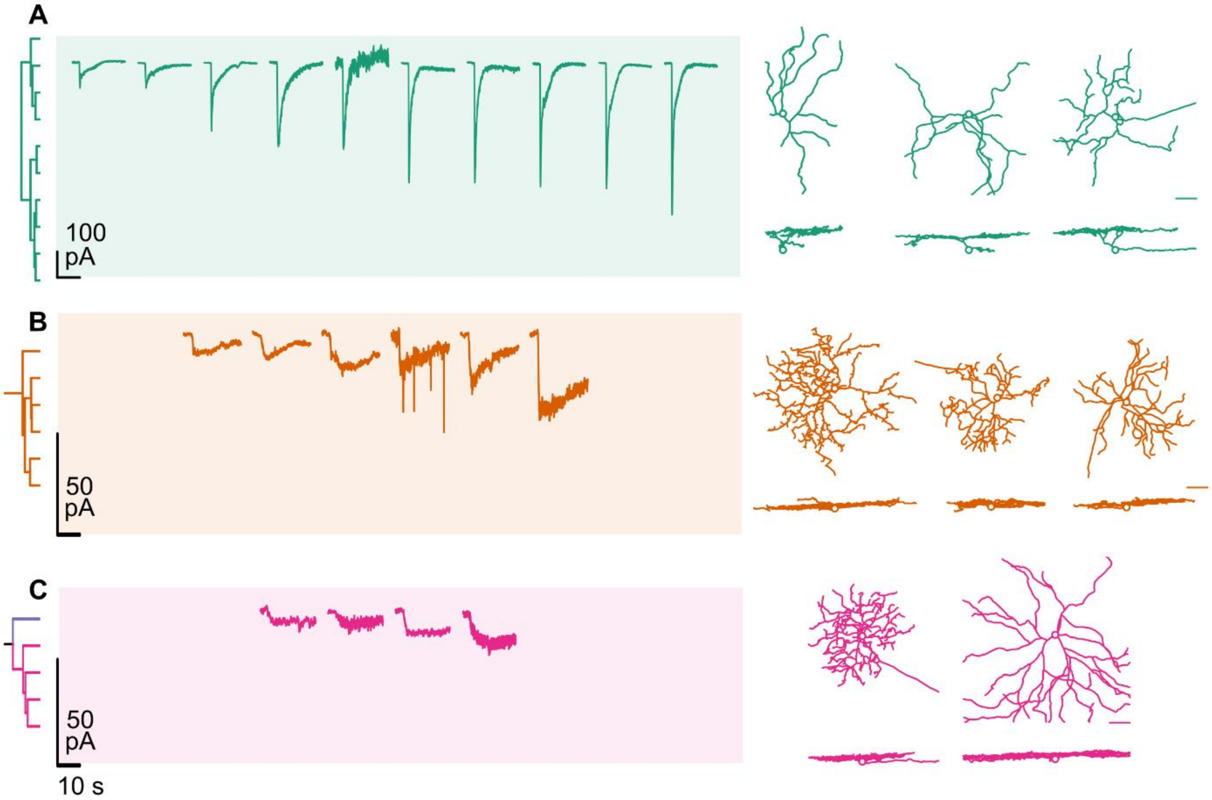

Supplemental Figure 4 | Diversity of intrinsic photocurrents and morphologies of cells in the *Opn4$^{cre(DSO)}$*; *Ai9* mouse retinam related to Figure 4.

(A-C) Voltage clamp recordings of intrinsic photocurrents (left) and dye-filled cells (right). Colors and dendrogram reflect clustering of currents highlighted in Figure 4. Animal age = Postnatal day 60 (P60).

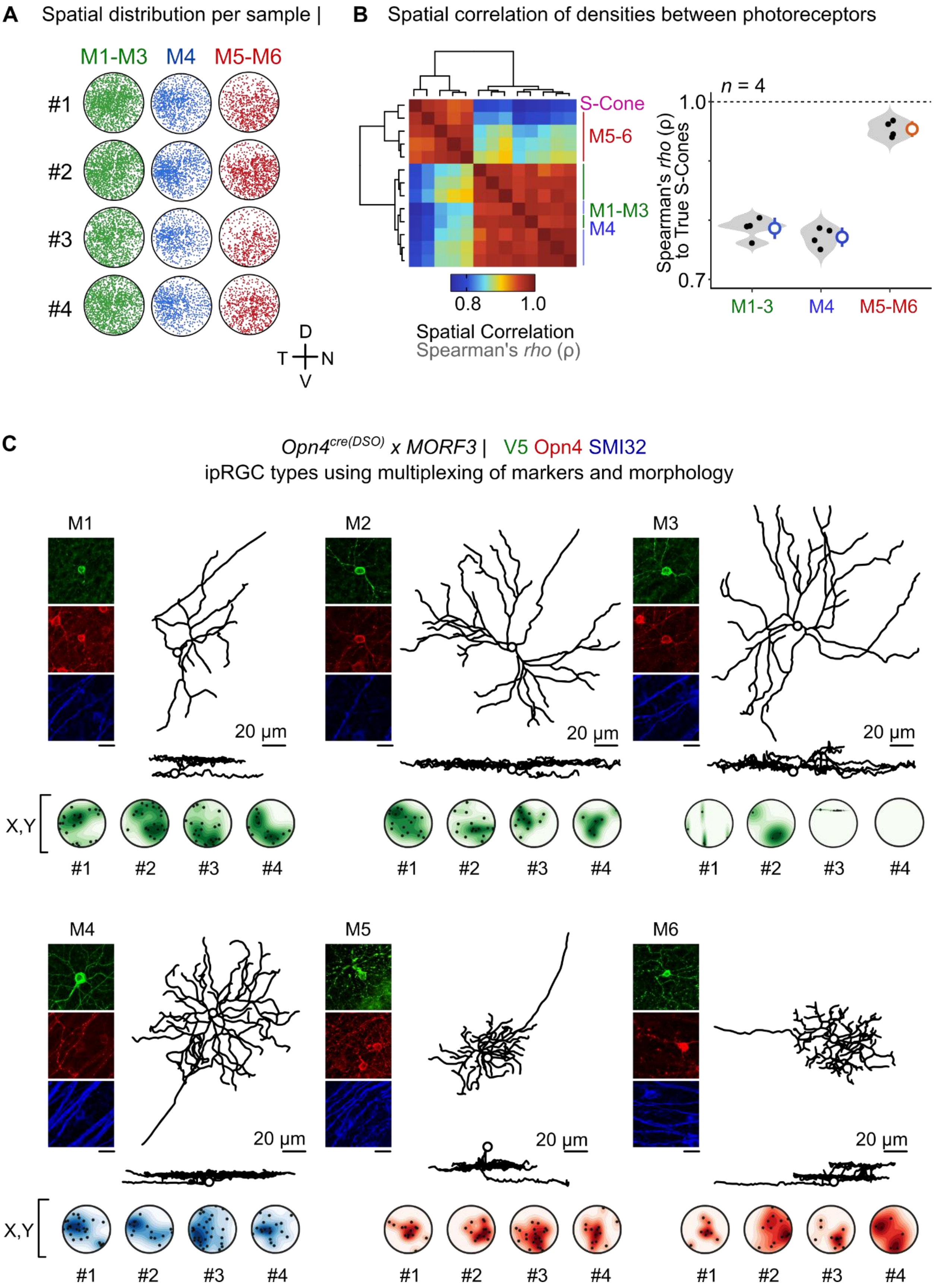
A Spatial distribution per sample |
M1-M3 M4 M5-M6
#1
#2
#3
#4
D
T N
V
B Spatial correlation of densities between photoreceptors
S-Cone
M5-6
M1-M3
M4
0.8 1.0
Spatial Correlation
Spearman's rho (ρ)
n = 4
1.0
0.7
Spearman's rho (ρ) to True S-Cones
M1-3 M4 M5-M6
C
Opn4cre(DSO) x MORF3 | V5 Opn4 SMI32
ipRGC types using multiplexing of markers and morphology
M1
M2
M3
20 μm
X,Y
#1 #2 #3 #4
M4
M5
M6
20 μm
X,Y
#1 #2 #3 #4

Supplemental Figure 5 | Spatial distributions and single-cell reconstructions with molecular marker multiplexing to identify ipRGC distributions across the retina, related to Figure 5.

(A) Individual polar plots of coarse ipRGC types (M1-M3 tdTomato+ Opn4+; M4 tdTomato+ SMI32+; M5-M6 tdTomato+ Opn4- SMI32-) in the *Opn4*$^{cre(DSO)}$*; Ai14* line. **(B)** Spearmann's correlation of normalized spatial densities of ipRGC types and True S-Cones. Each row and column in the heatmap represent an individual *n* polar plot. **(C)** Examples of ipRGC types reconstructed with cognate immunofluorescence of V5 (green), Opn4 (red), and SMI32 (blue), with individual polar plots of each type. Number refers to sample ID number. Animal age = Postnatal day 60 (P60).

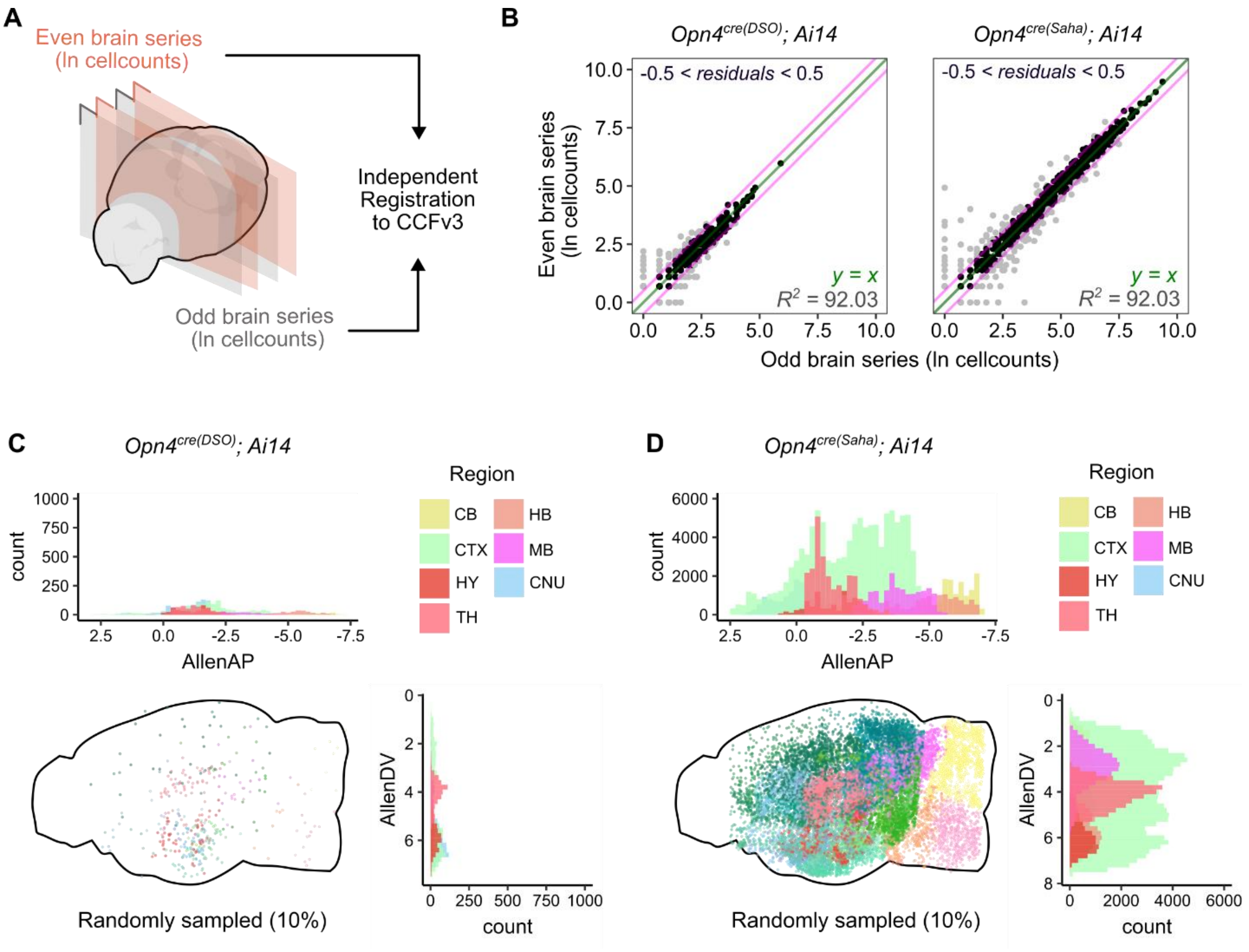

Supplemental Figure 6 | Registration pipeline validation and distribution of cells in the brains of *Opn4cre* mice, related to Figure 6.

(A) Schematic representation of registration validation performed on both *Cre* line sections. Series were split into alternating slices (Odd / Even brain series) and independently registered to the Allen CCFv3. **(B)** Joint linear correlation between odd and even series. Each point represents an individual brain region (~638), black dots represent residuals between -0.5 and 0.5. Green line represents a linear $y = x$ fit, magenta lines represent $y = x + 0.5$ and $y = x - 0.5$. **(C)** Profile view of cell distributions across the *Opn4*$^{cre(DSO)}$; *Ai14* brain. **(D)** Similar to **(C)** but in the *Opn4*$^{cre(Saha)}$; *Ai14* line. AllenAP = anterior-posterior axis (mm), AllenDV = dorsal-ventral axis (mm). Notice scale differences in y-axes in count. This was done to highlight cells in the *Opn4*$^{cre(DSO)}$ line, as the *Saha* scale would eclipse the histograms in the *DSO* plots. Animal age = Postnatal day 60 (P60).

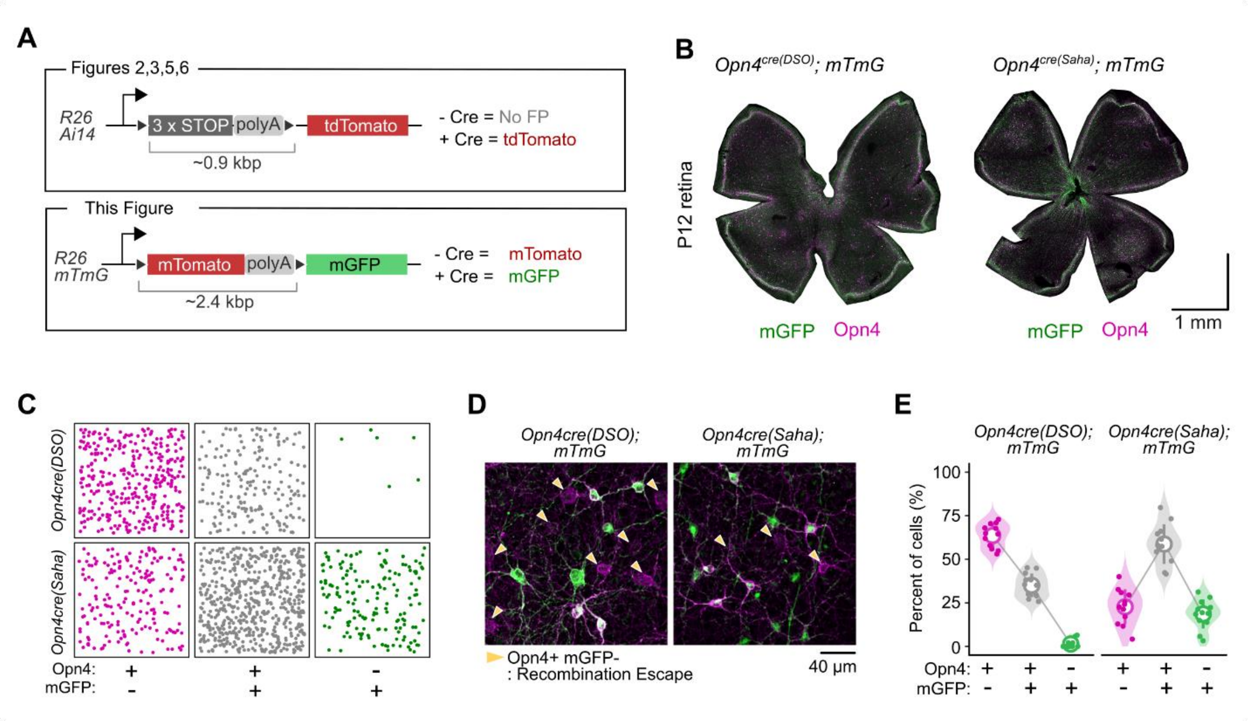

Supplemental Figure 7 | Reporter inter-*loxP* site distances augment recombination escape in *Opn4cre* lines, related to Figures 2-6. (A) Schematic depicting *Cre*-dependent reporters used in this study (top) and this figure (bottom). (B) Representative flat-mount retina from *Opn4cre(DSO)* and *Opn4cre(Saha*) crossed to *mTmG*. (C) Spatial point distributions of escaped cell (Opn4+ mGFP-; magenta), appropriately targeted cells (Opn4+ mGFP+; grey) and targeted cells (Opn4- mGFP+). (D) Representative images of analysis performed in (C & E). Arrowheads depict escaped cells (Opn4+ mGFP-). Mixed plots depicting percent of all analyzed cells in each category, across both lines. Small points = individual FOVs, large circles = mean of group, error bars = standard deviation. Animal age = Postnatal day 12 (P12).
